# Sequential Surgical Procedures in Vascular Surgery Patients Are Associated With Perioperative Adverse Cardiac Events

**DOI:** 10.3389/fcvm.2020.00013

**Published:** 2020-02-18

**Authors:** Henrike Janssen, Larissa Felgner, Laura Kummer, Hans-Jörg Gillmann, Claudia Schrimpf, Saad Rustum, Ralf Lichtinghagen, Bianca Sahlmann, Markus A. Weigand, Omke E. Teebken, Gregor Theilmeier, Jan Larmann

**Affiliations:** ^1^Department of Anesthesiology, University Hospital Heidelberg, Heidelberg, Germany; ^2^Department of Anesthesiology and Intensive Care Medicine, Hannover Medical School, Hanover, Germany; ^3^Department of Cardiothoracic, Transplantation and Vascular Surgery, Hannover Medical School, Hanover, Germany; ^4^Institute for Clinical Chemistry, Medical School Hannover, Hanover, Germany; ^5^Department of Human Medicine, Perioperative Inflammation and Infection, Faculty of Medicine and Health Sciences, University of Oldenburg, Oldenburg, Germany; ^6^Department of Anesthesiology, University Medical Center Groningen, Groningen, Netherlands

**Keywords:** perioperative inflammation, regulatory T cells, plaque vulnerability, cardiac adverse event, vascular surgery, interleukin-6

## Abstract

Patients at elevated cardiovascular risk are prone to perioperative cardiovascular complications, like myocardial injury after non-cardiac surgery (MINS). We have demonstrated in a mouse model of atherosclerosis that perioperative stress leads to an increase in plaque volume and higher plaque vulnerability. Regulatory T cells (Tregs) play a pivotal role in development and destabilization of atherosclerotic plaques. For this exploratory *post-hoc* analysis we identified 40 patients recruited into a prospective perioperative biomarker study, who within the inclusion period underwent sequential open vascular surgery. On the basis of protein markers measured in the biomarker study, we evaluated the perioperative inflammatory response in patients' plasma before and after index surgery as well as before and after a second surgical procedure. We also analyzed available immunohistochemistry samples to describe plaque vulnerability in patients who underwent bilateral carotid endarterectomy (CEA) in two subsequent surgical procedures. Finally, we assessed if MINS was associated with sequential surgery. The inflammatory response of both surgeries was characterized by postoperative increases of interleukin-6,−10, Pentraxin 3 and C-reactive protein with no clear-cut difference between the two time points of surgery. Plaques from CEA extracted during the second surgery contained less Tregs, as measured by Foxp3 staining, than plaques from the first intervention. The 2nd surgical procedure was associated with MINS. In conclusion, we provide descriptive evidence that sequential surgical procedures involve repeat inflammation, and we hypothesize that elevated rates of cardiovascular complications after the second procedure could be related to reduced levels of intraplaque Tregs, a finding that deserves confirmatory testing and mechanistic exploration in future populations.

## Introduction

Patients at elevated risk for cardiovascular complications are prone to perioperative adverse cardiac events, such as myocardial injury after non-cardiac surgery (MINS) (1) and face higher perioperative mortality (1–4). Atherosclerosis and its complications are driven by systemic inflammation (5), however the mechanisms underlying perioperative cardiovascular complications are not fully understood (6, 7). We and others have demonstrated that in mouse models of atherosclerosis, perioperative stress leads to a systemic inflammatory reaction resulting in a rapid, interleukin-6 (IL-6)-dependent, expansion of plaque volume and increase in plaque vulnerability (8, 9). Perioperatively released IL-6 might also have an important role in human plaque fissuring (10). In mice and man, unstable atherosclerotic lesions are prone to the development of plaque fissures and rupture (11, 12). Plaque fissures and ruptures are among the mechanisms underlying MINS (7). About 25% of patients with perioperative acute coronary synrome show signs of coronary plaque rupture (13).

Low numbers of regulatory T cells (Tregs) in blood, as well as in human atherosclerotic plaque, are associated with plaque vulnerability (14, 15) and modulating Treg numbers in mouse models of atherosclerosis regulates progression of atherosclerotic lesion development (16). Additionally, we have recently shown that preoperative low counts of Tregs are associated with higher rates of MINS and adverse cardiovascular outcome in patients with coronary heart disease undergoing non-cardiac surgery (17).

However, it is unknown if perioperative stress associated with surgical procedures renders patients prone to MINS. Also, it is unclear whether Treg numbers in atherosclerotic lesions as a measure of plaque stability are affected by surgical interventions.

Therefore, for this explorative *post-hoc* analysis we identified all patients from a perioperative vascular surgery biomarker study (18, 19), who within the study recruitment period underwent sequential vascular surgical procedures such as carotid endarterectomy (CEA). We described the systemic perioperative inflammatory response and quantified Tregs as a measure of plaque stability in available CEA samples from the first (1st) and second (2nd) intervention and compared MINS rates between the two sequential surgeries. We demonstrate that surgery is associated with cardiac complications in subsequent surgical procedures, and we provide descriptive evidence for the hypothesis that enhanced plaque vulnerability might be related to MINS.

## Materials and Methods

### Study Design and Selected Population

**Figure 1:**
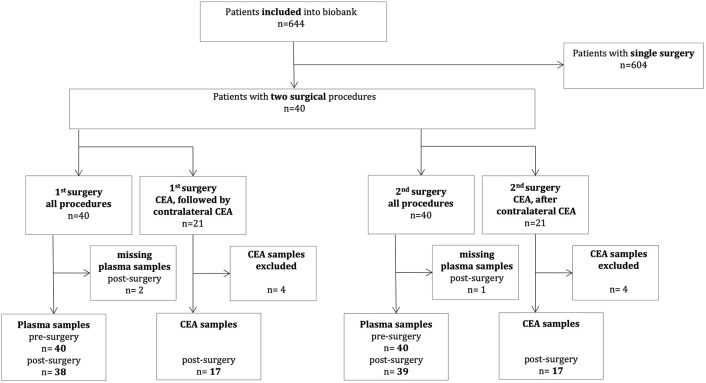
Patient flow chart. CEA, Carotid endarterectomy.

Details concerning study design and enrollment are reported in Gillmann et al. (18) and Schrimpf et al. (19). In brief, within a single-center, prospective, observational study aiming at identification of patients at risk for perioperative cardiovascular complications, 644 consecutive patients undergoing an elective open vascular procedure (aortic, peripheral artery or carotid surgery) in general anesthesia were prospectively recruited into a biomarker study. Endovascular procedures were not included. The study was performed between June 2007 and October 2012 at the Department of Cardiac, Thoracic, Transplantation and Vascular Surgery and the Department of Anesthesiology and Intensive Care Medicine at Hannover Medical School and was approved by the Hannover Medical School's Ethics Committee (approval no. 4598, 14/MAY/2007). Written informed consent was obtained from all patients prior to enrollment into the biomarker study. Patients were followed up for the occurrence of major cardiovascular events until the 30th postsurgical day, pre- and postoperative day-1 plasma samples as well as available endarterectomy samples were collected as published before (18, 19). In this exploratory *post-hoc* analysis, we included all 40 patients who underwent two sequential surgical procedures within the recruitment period. Patient flow and availability of patients' plasma and CEA samples are depicted in Figure 1. After the 1st surgery, two postoperative plasma samples and after the 2nd surgery one postoperative plasma sample were lost because of technical difficulties. For histology analyses, we identified 21 patients who underwent bilateral CEA in the inclusion period. These patients were screened for histological analysis for direct comparison for each patient between the sequential procedures. Damaged samples were excluded, leaving 17 patients being selected for histological assessment with two samples per patient (Figure 1).

MINS was defined as any increase in postoperative high-sensitive cardiac troponin T (hs-cTnT) ≥50 ng l^−1^ (20) judged due to myocardial ischemia. Hs-cTnT rise was defined as an increase of at least 50% from baseline (21). Troponin elevations were judged to be due to myocardial injury if no evidence for primarily extracardiac diseases such as sepsis or pulmonary embolism was observed.

### Biochemical Analysis in Plasma

Blood samples for biomarker measurements were collected immediately before surgery and on day one after surgery. Plasma was stored at −80°C and measurements were performed in batch assays. Hs-cTnT levels were measured preoperatively and postoperatively (Hs-cTnT; Roche Diagnostics, Mannheim, Germany; Elecsys® 2010/ cobasTM e411 immunoanalyser). A commercially available enzyme-linked immunosorbent assay (ELISA) was used to measure plasma levels of Pentraxin 3 (PTX3) (R&D Systems, Minneapolis, USA). Plasma levels of IL-6 and Interleukin-10 (IL-10) were measured by a cytometric bead array (CBA) (BD Biosciences, Franklin Lakes, USA). C-reactive protein (CRP) measurements were pulled from patients' regular perioperative blood work which were performed in the hospital's laboratory for routine measurements.

### Histological Analysis on Carotid Artery Plaque

From patients who underwent procedures with endarterectomy, samples were collected and cryoembedded in Tissue Tek O.C.T. Serial cryosections from available CEA were cut at a thickness of 5 μm. Series of 25 serial slides were prepared. The first 25 cryosections were each placed on position one of the 25 slides. The next 25 sections were placed on position two and the third 25 slides were placed on position three. Thus, on each cryosection the distance from slice one to two and from two to three was 125 μm. In total, each microscopic slide represented a distance of 250 μm. Sections were stained for Hematoxylin & Eosin (H&E) and then subjected to further immunohistochemical analyses. For each sample we identified the most advanced part of the plaque, which was used for immunohistochemical analyses. For each staining, 3 slides (in total 9 sections) were analyzed (Supplementary Figure 1). Double fluorescent immune histochemical stainings were performed for macrophages (mouse anti-human CD68, clone KP1, DAKO, Santa Clara, USA) and collagen (rabbit anti-human collagen I, Chemicon/Merck Millipore, Burlington, USA), as well as α-vascular smooth muscle cells (VSMC) (mouse anti-human SMA, clone 1A4, DAKO, Santa Clara, USA) and endothelium (rabbit anti-human von willebrand factor, DAKO, Santa Clara, USA). In addition, immunhistochemical stainings for glycophorin A (mouse anti-human CD235a, DAKO, Santa Clara, USA), CD4 (mouse anti-human CD4, clone RPA-T4, Invitrogen, Waltham, USA) and for FOXP3 (mouse anti-human FOXP3, Abcam, Cambridge, UK) were performed. Samples were classified by two independent investigators and are based on the modified American Heart Association (AHA) Classification by Virmani et al. (22).

### Statistical Analysis

Continuous data were compared using Wilcoxon matched-pairs signed rank test. Categorical variables are expressed as absolute and relative counts and were compared using Chi-square test. Plasma measurements were compared by Kruskal–Wallis test for global assessment of differences in the data family before limited group wise comparisons were done using Dunn's test. To account for multiple comparisons, statistical analyses of plasma were adjusted according to Bonferroni (α <0.05/4). Histological measurements were compared by Wilcoxon matched-pairs signed rank test. Incidence of MINS was compared using Chi-square test. Data are presented as median [interquartile ranges (IQR)], unless otherwise stated, with whiskers extending from 5th to 95th percentile. Descriptive *p*-values are reported.

## Results

### Baseline Characteristics

Clinical and demographical baseline characteristics for included patients in relation to the time point of 1st and 2nd surgery are presented in Table 1. Median age was 70 years and 75% of patients were of male gender. The study population presented with a high burden of cardiovascular disease. Consequently, the great majority received cardiovascular medication. Overall there were no changes in patients' cardiovascular related health between the two time points. CEA was the most common open vascular procedure performed. Patients did not spend more time in hospital or intensive care compared between the two procedural time points (Table 1). The median time between the two surgical procedures was 51 days (51 [47; 96] days).

**Table 1:** Baseline characteristics of patients.

**Variable**	**1st surgery**	**2nd surgery**	***p-value***
	**Total (*n* = 40)**	**Total (*n* = 40)**	
Age; years	71 (65; 75)	71 (66; 76)	
Male sex; n (%)	30 (75)	30 (75)	
Hospital days	7 (5, 9)	6 (5, 10)	>0.05
ICU days	1 (0; 1)	1 (0; 1)	>0.05
**Type of surgical procedure**, ***n*** **(%)**			
Carotid	26 (65)	23 (58)	>0.05
Peripheral artery	9 (23)	14 (35)	>0.05
Aortic	5 (13)	3 (8)	>0.05
**Medical history**, ***n*** **(%)**			
Arterial hypertension	34 (85)	35 (88)	>0.05
Diabetes mellitus	12 (30)	12 (30)	>0.05
CAD or MI	16 (40)	17 (43)	>0.05
Stroke or TIA	11 (28)	13 (33)	>0.05
Heart failure	4 (10)	5 (13)	>0.05
**Preoperative medication**, ***n*** **(%)**			
ASS	29 (73)	31 (78)	>0.05
ADP receptor antagonist	4 (10)	3 (8)	>0.05
Beta blockers	24 (60)	23 (58)	>0.05
Statins	28 (70)	29 (73)	>0.05
AT II blockers	9 (23)	9 (23)	>0.05
ACE inhibitors	27 (68)	21 (53)	>0.05
Calcium antagonists	17 (43)	16 (40)	>0.05
Diuretics	20 (50)	21 (53)	>0.05

*Data are presented as median (interquartile range) or as absolute number (percentage). Continuous data were compared using Wilcoxon matched-pairs signed rank test. Categorical variables were compared using Chi-square test. ICU, intensive care unit; CAD, coronary artery disease; MI, myocardial infarction; TIA, transient ischemic attack; AT angiotensin; ACE angiotensin converting enzyme*.

### Perioperative Systemic Inflammation

**Figure 2:**
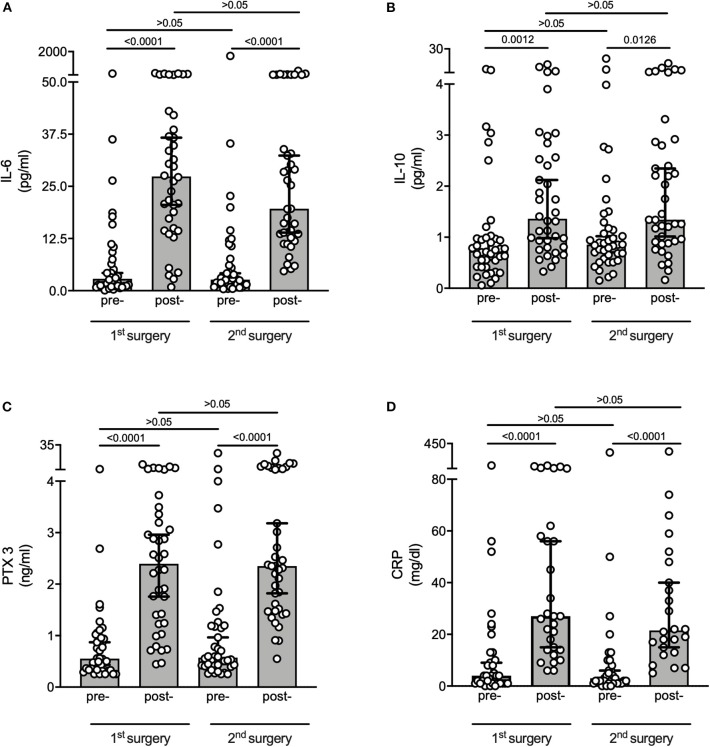
Perioperative systemic inflammation. Measurements were performed in pre- and postoperative plasma samples of 1st and 2nd surgery. **(A)** IL-6 measured by CBA, **(B)** IL-10 measured by CBA, **(C)** PTX3 measured by ELISA, **(D)** CRP taken from routinely performed perioperative blood work. Graphs are displayed as median with whiskers extending from 5th to 95th percentile. Data were compared using Kruskal–Wallis test for global assessment of differences in the data family before limited group wise comparisons were done using Dunn's test. To account for multiple comparisons, statistical analyses of plasma were adjusted according to Bonferroni (α <0.05/4). Overall, both surgeries led to a similar inflammatory response associated with detection of markers for increased plaque vulnerability. IL, Interleukin; PTX3, Pentraxin 3; CRP, C-reactive protein.

For assessment of the perioperative inflammatory response, we measured plasma levels of IL-6, IL-10 and PTX3 prior to and after 1st and 2nd surgery. Additionally, we pulled patients CRP levels from routinely performed bloodwork prior to and after 1st and 2nd surgery. Comparing pre- to post-surgical measurements, plasma levels of IL-6 increased after both surgeries [(1st procedure pre- 2.84 [1.16; 7.23] pg/ml vs. post- 27.38 [14.82; 42.32] pg/ml, *p* < 0.0001) (2nd procedure pre- 2.64 [1.51; 7.25] pg/ml vs. post- 19.62 [12.73; 53.74] pg/ml, *p* < 0.0001)] (Figure 2A). Also plasma levels for IL-10 increased after both surgical procedures compared to pre-surgery endpoints [(1st procedure pre- 0.73 [0.41; 0.98] pg/ml vs. post- 1.36 [0.81; 2.63] pg/ml, *p* = 0.0012) (2^nd^ procedure pre- 0.86 [0.60; 1.26] pg/ml vs. post- 1.34 [0.91; 2.87] pg/ml, *p* = 0.0126)] (Figure 2B). PTX3 increased after both surgeries, comparing pre- to postoperative plasma levels [(1st procedure pre- 0.55 [0.33; 0.99] ng/ml vs. post- 2.39 [1.24; 3.39] ng/ml, *p* < 0.0001) (2nd procedure pre- 0.57 [0.42; 1.19] ng/ml vs. post- 2.35 [1.48; 5.91] ng/ml, *p* < 0.0001)] (Figure 2C). CRP increased after both surgical procedures compared to preoperative values [(1st procedure pre- 4 [1; 13] mg/dl vs. post- 27 [14; 60] mg/dl, *p* < 0.0001) (2nd procedure pre- 1 [1; 9] mg/dl vs. post- 21.5 [13.5; 46] mg/dl, *p* < 0.0001)] (Figure 2D). Pre-surgical values prior to the 2nd procedure did not differ from measurements prior to the 1st intervention. Neither were post-surgical values after the 2nd procedure different from values after the 1st intervention (Figures 2A–D).

### Histological Assessment of Atherosclerotic Lesions From Carotid Endarterectomy

**Figure 3:**
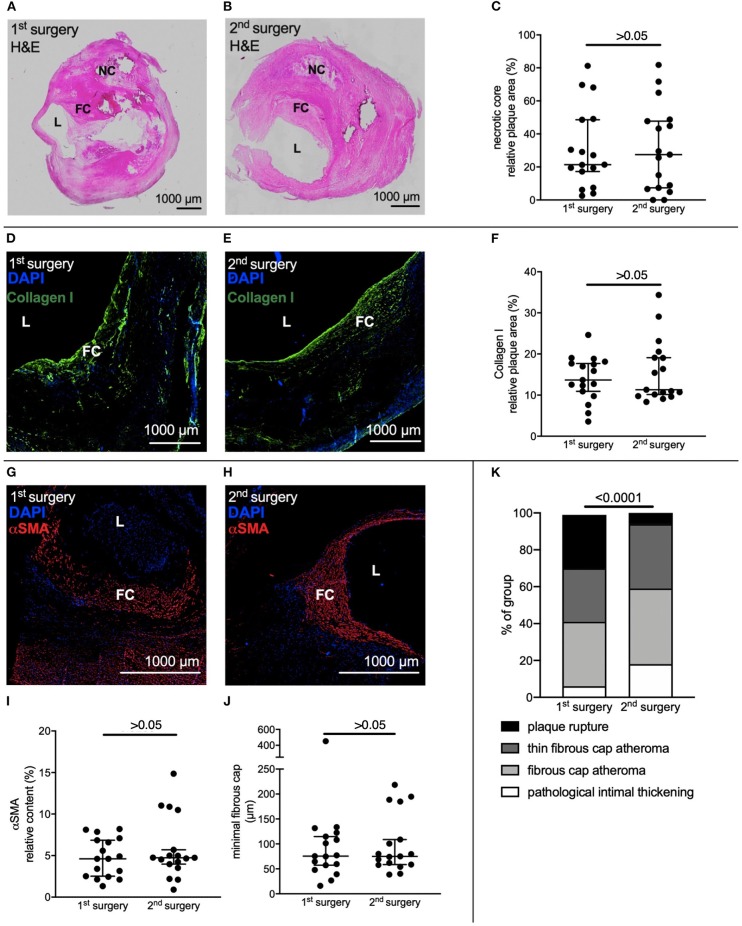
Histological assessment of atherosclerotic lesions from carotid endarterectomy. CEA samples of 17 patients who underwent bilateral CEA were selected for histological assessment. **(A,B)** Representative H&E staining of CEA samples from the same patient of 1st and 2nd surgery. **(C)** Relative necrotic core size did not differ between 1st and 2nd CEA sample. **(D,E)** Representative immune fluorescent staining of Collagen I of CEA samples from the same patient of 1st and 2nd surgery **(F)** Relative Collagen I content did not differ. **(G,H)** Representative immune fluorescent staining of αSMA of CEA samples from the same patient of 1st and 2nd surgery. **(I,J)** Relative content of αSMA and minimal thickness of fibrous cap did not differ. **(K)** Virmani Classification of samples harvested at both surgeries. Overall, plaques were more advanced in the 1st surgical cohort. **(C,F,I,J)** Wilcoxon matched-pairs signed rank test. Data is presented as median with whiskers extending from 5th to 95th percentile. **(K)** Chi-square test. H&E, Hematoxylin & Eosin; L, Lumen, NC, Necrotic core; FC, Fibrous cap; αSMA, alpha-smooth muscle actin.

Samples from CEA were stained for H&E (Figures 3A,B). Size of necrotic core did not differ comparing CEA from 1st to CEA from 2nd surgery (1st 27.16 [13.46; 48.85] % vs. 2nd 25.64 [7.05; 48.24] %, *p* > 0.05) (Figure 3C). Collagen content was judged on staining of Collagen I (Figures 3D, E), which did not differ comparing CEA from 1st to CEA from 2nd surgery (1st 13.68 [10.33; 17.92] % vs. 2nd 11.30 [9.96; 19.83] %, *p* > 0.05) (Figure 3F). Samples were stained for α-smooth muscle actin (αSMA) for detection of VSMC (Figures 3G,H). Relative content of αSMA did not differ between 1st and 2nd surgery (1st 4.62 [2.49; 6.97] % vs. 2nd 4.75 [3.75; 8.10] %, *p* > 0.05) (Figure 3I) Minimal fibrous cap thickness also did not differ between samples from 1st to 2nd surgery (1st 75.63 [52.72; 118.7] μm vs. 2nd 75.15 [58.15; 146.90] μm, *p* > 0.05) (Figure 3J). Samples were then stratified into groups based on the modified AHA Classification published by Virmani et al. (22). Overall, lesions were of an intermediate to advanced stage. We chose the classification by Virmani, as it focuses on AHA classification IV to VI (22). Atherosclerotic lesions from the 1st CEA more frequently showed plaque ruptures and less pathological intimal thickening, classifying plaques overall as more advanced (*p* < 0.001) (Figure 3K).

### Quantification of Leukocyte Subpopulations in Atherosclerotic Lesions From Carotid Endarterectomy

**Figure 4:**
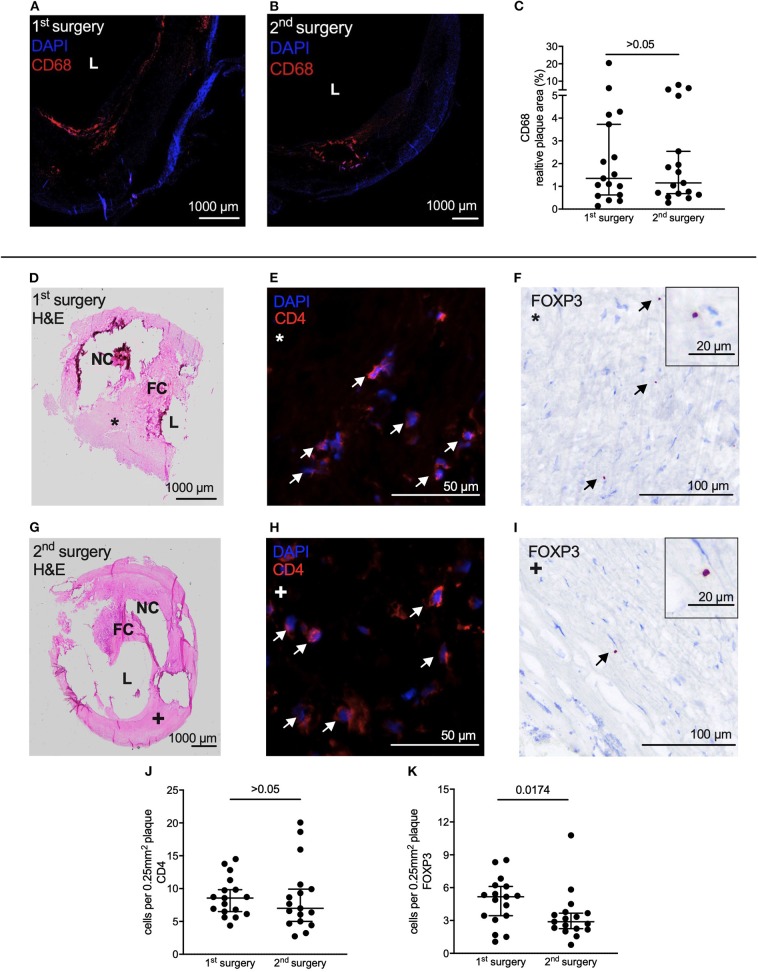
Assessment of plaque vulnerability based on leukocyte subpopulation content. **(A,B)** Representative immune fluorescent staining of CD68 of CEA samples from the same patient of 1st and 2nd CEA. **(C)** Relative content of CD68 did not differ between 1st and 2nd CEA sample. **(D)** H&E staining of the sample from the 1st CEA was used for identification of plaque shoulders. **(E)** Representative staining of CD4 and **(F)** of FOXP3 on a patient's sample from the 1st CEA. Arrows indicate CD4 and FOXP3 positive cells, respectively. **(G)** H&E staining of the same patient on the sample from the 2nd CEA. **(H)** Representative staining of CD4 and **(I)** FOXP3 on the sample from the 2nd CEA. Arrows indicate positive cells. **(J)** Content of CD4 positive cells did not differ between 1st and 2nd CEA sample in areas identified as plaque shoulders. **(K)** Samples from the 2nd surgery contained less FOXP3 positive cells in plaque shoulders. **(C,J,K)** Wilcoxon matched-pairs signed rank test. Data is presented as median with whiskers extending from 5th to 95th percentile. L, Lumen; H&E, Hematoxylin & Eosin; NC, Necrotic core; FC, Fibrous cap.

Staining for CD68 resulted in no difference in relative content between the two groups (1st 1.35 [0.60; 3.94] % vs. 2nd 1.15 [0.66; 3.76] %, *p* > 0.05) (Figures 4A–C). For quantification of CD4 and FOXP3 positive cells, we adopted the quantification method by Dietel et al. (15). Two regions with the size of 0.25 mm^2^ per section were identified in plaque shoulders in synopsis with corresponding H&E stainings and then analyzed for CD4 and FOXP3 positive cells, respectively (Figures 4D–I). There was no difference in content of CD4 positive cells between 1st and 2nd surgery (1st 8.56 [6.32; 10.56] cells/0.25 mm^2^ vs. 2nd 7.00 [4.84; 10.27] cells/0.25 mm^2^, *p* > 0.05) (Figure 4J). However, less FOXP3 positive cells were detected in the samples from the 2nd surgery (1st 5.17 [3.25; 6.17] cells/0.25 mm^2^ vs. 2nd 2.89 [2.21; 3.73] cells/0.25 mm^2^, *p* = 0.0174) (Figure 4K).

### Incidence of Myocardial Injury After First and Second Surgical Procedure

**Figure 5:**
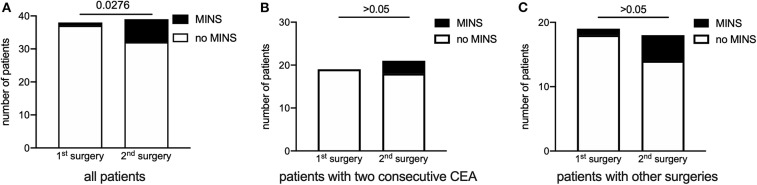
Incidence of myocardial injury after first and second surgical procedure. **(A)** After the 1st surgery one patient was diagnosed with MINS, after the 2nd surgery seven patients suffered MINS. The 2nd surgical procedure was associated with MINS (OR 8.1, 95% CI [1.3; 93.3]). **(B)** In the subgroup of patients undergoing consecutive CEA, no patient suffered MINS after the first procedure and three after the second. **(C)** Display of patients with any other combination of surgery. One patient was diagnosed with MINS after the first procedure and four after the second. Chi-square test. Data are presented as absolute numbers per group. CEA, Carotid endarterectomy.

In patients suffering MINS median hs-cTnT value before surgery was 29.83 ng l^−1^ [12.60; 55.70], min. 7.67, max. 74.16 ng l^−1^ and 58.10 ng l^−1^ [27.46; 77.33], min. 13.19, max. 316.5 ng l^−1^ after the surgery. In the group without MINS median hs-cTnT value before surgery was 12.40 ng l^−1^ [7.67; 27.60], min. 3, max. 288 ng l^−1^ and 14.91 ng l^−1^ [8.54; 29.65], min. 3, max. 307.4 ng l^−1^ after the surgery. Incidence of MINS in the whole cohort differed between 1st and 2nd surgical intervention. After the 1st surgery one patient was diagnosed with MINS, after the 2nd surgery seven patients suffered MINS, demonstrating an increased risk after the 2nd procedure (1 vs. 7 patients, 1st vs. 2nd surgery, OR 8.1, 95% CI [1.3; 93.2], *p* = 0.0276) (Figure 5A). When taking into account only patients with consecutive CEA, no patients suffered MINS after the first procedure and three after the second (Figure 5B). In the subgroup of patients with other surgeries, one patient was diagnosed with MINS after the first procedure and four after the second (Figure 5C).

## Discussion

In this *post-hoc* analysis we demonstrate that in our cohort of patients at elevated cardiovascular risk undergoing vascular surgery, reoperation was associated with an increased MINS incidence. We analyzed atherosclerotic lesions from a subgroup of patients who underwent CEA twice. In those patients we found reduced Treg infiltration in 2nd surgery samples as a sign of higher atherosclerotic plaque vulnerability. This finding was independent from the incidence of MINS. There were no differences in patients' baseline characteristics. We did not detect a difference in the pattern of measured plasma markers (IL-6, IL-10, PTX3, CRP) between the first and second surgery. Surgery was associated with an increase in IL-6 postoperatively. High levels of postoperative IL-6 have proven to be an indicator for adverse outcome after vascular and abdominal surgery (23) and might hold a direct effect on plaque stability (10). This is also in line with our finding that mice develop bigger and more vulnerable plaques when exposed to perioperative stress in an IL-6 dependent manner (8). Because perioperative inflammation always consists of a balance between a pro- and anti-inflammatory response, we measured IL-10 as an immunosuppressant cytokine and also detected a postoperative increase. Postoperative elevation of IL-10 has been well-described (24). The surgical impact also was associated with an increase in postoperative PTX3 and CRP after both surgeries. PTX3, like CRP, belongs to the superfamily of pentraxins (25). In contrast to CRP it is not of hepatic origin, rather than for instance secreted by vascular endothelial cells and monocytes at the site of inflammation, e.g., atherosclerotic lesions (25). As a perioperative marker of inflammation, changes in PTX3 plasma levels are subtle compared to CRP (25). We expected small changes in the perioperative period. Recently several studies have shown that elevated PTX3 plasma levels are an indicator for atherosclerotic plaque vulnerability (26–28). Therefore, surgical-associated inflammation, like the observed PTX3 increase in plasma, could have an impact on local inflammatory processes in atherosclerotic lesions.

Overall, we established that patients in our study exposed to two surgical hits experienced increases in circulating pro- and anti-inflammatory cytokines. Importantly, direct comparison of the inflammatory response between both surgeries did not differ, leaving the possibility of a stronger systemic inflammatory response to the reoperation unlikely.

Histological assessment of atherosclerotic lesions from patients, who underwent CEA twice, showed that necrotic core size did not differ. Based on the fact that patients with symptomatic carotid artery stenosis have larger necrotic core sizes, we anticipated that there might be a difference in necrotic core size (29). Also plaque progression, mainly through an increase in necrotic core size, happens shortly before myocardial infarction (30) and perioperative stress in mice is associated with an increase in necrotic core size (8). Relative content of collagen I did not differ between samples from 1st to 2nd surgery. Collagen holds a particular role in plaque stability related to shear stress (31) and adhesion of the lesion to the vessel wall (32). Low minimum cap thickness is associated with plaque rupture (33) but relative content of VSMC, as well as minimal thickness of fibrous cap was not different between samples of 1st and 2nd surgery. When stratified according to the classification by Virmani (22), CEA samples from the 1st surgery more frequently showed plaque ruptures. This is in line with the expectation that a vascular surgeon would choose the site with higher stenosis, lesion complexity, and plaque vulnerability first for surgical intervention.

Plaques from the 2nd surgery did not contain more macrophages, quantified by CD68. Macrophages actively drive development and progression of atherosclerotic lesions and increase plaque vulnerability (34). IL-6 is a potential driver of adhesion and transmigration of macrophages (35). As migration processes of monocytes and macrophages in atherosclerosis have been well-established (36), we hypothesized that perioperative stress would lead to a higher influx of macrophages into the atherosclerotic lesions. This could not be confirmed. However, findings are in line with results from both mouse models of perioperative stress, which also did not show an increase in macrophages within the atherosclerotic plaque (8, 9).

Low levels of CD4 cells in plaques are associated with unstable atherosclerotic lesions (37). However, contrary to our expectation, content of CD4 positive cells did not differ between samples from 1st and 2nd surgery. CD4 positive cell differentiate into various subtypes of helper T cells which have been attributed pro- or anti-inflammatory in atherosclerosis (38). Accordingly, we quantified Treg content. Patients' CEA samples harvested after the 2nd surgery contained less Tregs compared directly to samples from the 1st surgery. Low numbers of circulating Tregs are associated with atherosclerosis (39). Additionally, we have recently shown that preoperative low counts of Tregs are associated with cardiac adverse events after non-cardiac surgery (17). When looking at the atherosclerotic lesion itself, low numbers of Tregs within the plaque have been associated with plaque vulnerability (15, 37). For the current study peripheral blood Treg counts are not available. Therefore, numbers cannot be compared to histological findings in this cohort. However, our previous results (17) in connection with the current findings lead to the intriguing hypothesis that there is a connection between circulating Tregs, plaque stability and patients' outcome after surgery. Oxidative stress increases Treg apoptosis in a high fat mouse model (40) rendering this also a possible mechanism explaining the lower Treg numbers in the plaques under perioperative stress from 2nd surgery. As Tregs produce IL-10 (39) one could expect to see differences in the IL-10 response to the 2nd surgical procedure. However, Tregs are not necessarily the origin of postoperative systemically secreted IL-10, as it has been recently shown that a postoperative increase of IL-10 mRNA in patients' blood was not accompanied with FOXP3 mRNA increase but in fact a decrease (24).

The 2nd surgical procedure was associated with MINS (OR 8.1). This suggests that not only in mice (8) but also in humans the perioperative inflammatory reaction is associated with cardiovascular events and on top of that might have an additive effect with repetitive surgical procedures. At this point, we cannot provide sufficient confirmative evidence to conclude that a lower in-plaque content of Tregs leads to higher plaque vulnerability. However, whilst acknowledging that MINS can be the consequence of diverse events in the perioperative phase, our results will stimulate further research and allow formulating the intriguing hypothesis that perioperative reduction of Treg content in atherosclerotic lesions might contribute to cardiovascular complications (10).

Our study has several limitations. We would like to highlight that only 70% of patients were on a perioperative statin treatment after 1st surgery. After 2nd surgery, surprisingly this number only increased to 73%, which does not represent current practice based on AHA and ESC guidelines (4, 41). However, inclusion period for this study was 2007 until 2010. The investigated population in the *post-hoc* analysis is small, and results are therefore rather exploratory than confirmatory of the hypothesis that perioperative stress alters Treg content in atherosclerotic lesions leading to higher plaque vulnerability and therefore MINS. Plaque samples were not available for all patients. However, to our knowledge we are the first to present a direct comparison of atherosclerotic lesions before and after a surgical impact within the same patient.

A prospective randomized trial to test if perioperative stress alters plaque stability in humans with regard to histological instability criteria is difficult to design. We performed a sample size calculation (G^*^Power 3) to estimate the number of patients necessary for a prospective study. We calculated with a more conservative odds ratio of 2, than 8.1, which we found in this preliminary study. Based on this assumption, 240 patients are necessary to find the expected difference in MINS vs. no MINS between 1st and 2nd surgery with a power (1 − β) of 0.8 and α = 0.05 (Two dependent groups Mc Nemar test). Keeping in mind that we identified 40 patients with two sequential vascular surgeries in a 5-year inclusion period out of a total of 644 included patients, highlights the difficulty of a prospective trial. Therefore, this analysis of an observational study is a reasonable alternative. Availability of atherosclerotic lesions from coronary vessels for histological characterization is limited, making samples from CEA a viable option. MINS is not exclusively a result of high plaque vulnerability leading to plaque rupture but can also be a result of an oxygen supply-demand mismatch (7, 42). We therefore are not able to conclude from our findings that the results are necessarily related. However, both findings show independently that perioperative inflammation has a negative impact on preexisting cardiovascular disease that worsens with reoccurring exposure.

In conclusion, we are the first to show that sequential surgeries are associated with MINS in patients undergoing open vascular surgery. Both surgical procedures led to an inflammatory response, whilst we could not detect any differences for inflammatory plasma markers between the two time points of surgery. Plaque samples from the 2nd procedure contained less Tregs, which might be an indicator for higher plaque vulnerability. Perioperative changes of Treg content in atherosclerotic lesions are of interest to shine further light on the impact of perioperative inflammation, especially in patients at elevated cardiovascular risk.

## Data Availability Statement

The datasets generated for this study are available on request to the corresponding author.

## Ethics Statement

The studies involving human participants were reviewed and approved by Ethics Committee Hannover Medical School, approval No. 4598, 14/MAY/20. The patients/participants provided their written informed consent to participate in this study.

## Author Contributions

HJ, H-JG, CS, SR, BS OT, GT, and JL collected data and samples. RL performed measurements of plasma biomarkers. GT, OT, H-JG, HJ, and JL designed the project. HJ, LF, LK, BS, and MW performed experiments and analyzed data. HJ, LF, LK, MW, and JL interpreted data. HJ, LF, and JL drafted the manuscripts. HJ, LK, H-JG, CS, SR, GT, and JL revised the manuscript. All authors read and approved the final manuscript.

### Conflict of Interest

The authors declare that the research was conducted in the absence of any commercial or financial relationships that could be construed as a potential conflict of interest.
