# Supplementary figures and images for: Sequential Surgical Procedures in Vascular Surgery Patients Are Associated With Perioperative Adverse Cardiac Events
Source: Front Cardiovasc Med. 2020 Feb 18;7:13. doi: 10.3389/fcvm.2020.00013 (PMC7040239; doi:10.3389/fcvm.2020.00013)

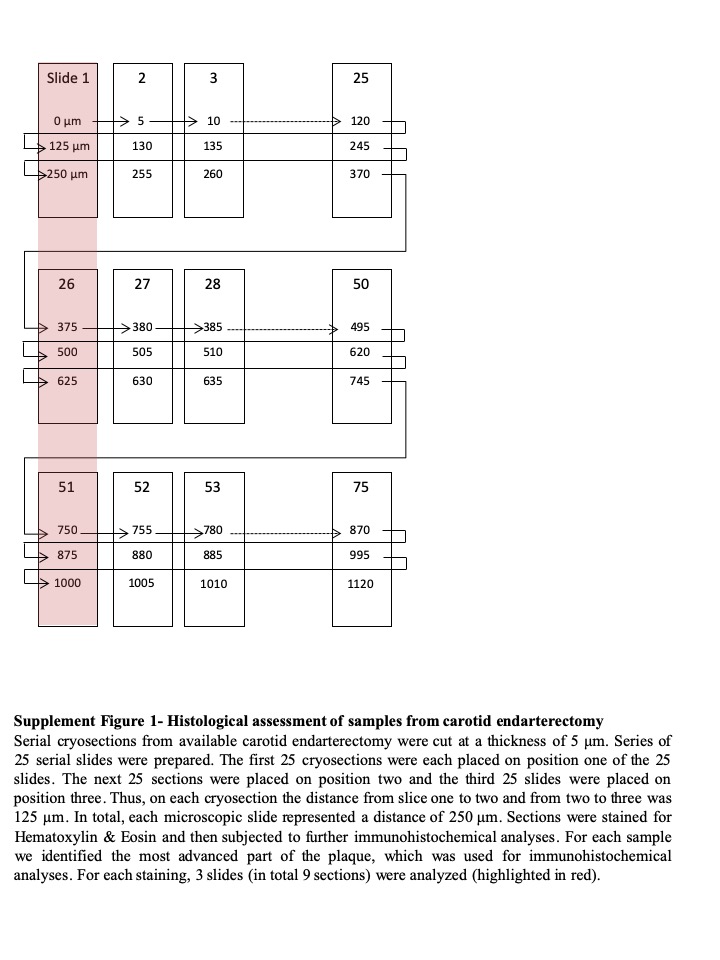

Supplement: Supplementary file 1 [file Image_1.JPEG]
